# Cochlin Deficiency Protects Aged Mice from Noise-Induced Hearing Loss

**DOI:** 10.3390/ijms222111549

**Published:** 2021-10-26

**Authors:** Dorien Verdoodt, Noa Peeleman, Krystyna Szewczyk, Guy Van Camp, Peter Ponsaerts, Vincent Van Rompaey

**Affiliations:** 1 Department of Translational Neurosciences, Faculty of Medicine and Health Sciences, University of Antwerp, 2610 Wilrijk, Belgium; noapeeleman@hotmail.com (N.P.); krystyna.szewczyk@uantwerpen.be (K.S.); vincent.vanrompaey@uantwerpen.be (V.V.R.); 2 Laboratory of Experimental Hematology, Faculty of Medicine and Health Sciences, Vaccine and Infectious Disease Institute (Vaxinfectio), University of Antwerp, 2610 Wilrijk, Belgium; peter.ponsaerts@uantwerpen.be; 3 Centre of Medical Genetics, Faculty of Medicine and Health Sciences, University of Antwerp, 2650 Edegem, Belgium; guy.vancamp@uantwerpen.be; 4 Department of Otorhinolaryngology and Head & Neck Surgery, Antwerp University Hospital, 2650 Edegem, Belgium

**Keywords:** noise exposure, inner ear inflammation, *Coch* knockout, spiral ligament

## Abstract

Several studies have shown that type IV fibrocytes, located in the spiral ligament, degenerate first after noise exposure. Interestingly, this is the region where *Coch* expression is most abundant. As it is suggested that cochlin plays a role in our innate immune system, our goal is to investigate hearing thresholds and inner ear inflammation after noise exposure in *Coch* knockout (*Coch^−/−^*) mice compared to *Coch* wildtype (*Coch^+/+^*) mice. Animals were randomly allocated to a noise exposure group and a control group. Vestibular and auditory testing was performed at 48 h and one week after noise exposure. Whole mount staining and cryosectioning of the cochlea was performed in order to investigate hair cells, spiral ganglion neurons, inner ear inflammation, *Coch* expression and fibrocyte degeneration. Hearing assessment revealed that *Coch^+/+^* mice had significantly larger threshold shifts than *Coch^−/−^* mice after noise exposure. We were unable to identify any differences in hair cells, neurons, fibrocytes and influx of macrophages in the inner ear between both groups. Interestingly, *Coch* expression was significantly lower in the group exposed to noise. Our results indicate that the absence of *Coch* has a protective influence on hearing thresholds after noise exposure, but this is not related to reduced inner ear inflammation in the knockout.

## 1. Introduction

Exposure to loud noise can lead to a decreased hearing function and tinnitus due to damage to both sensory and non-sensory cells in the inner ear [1]. Interestingly, the region that is most sensitive to noise exposure is the inferior region of the spiral ligament where the type IV fibrocytes are located, and *COCH* expression is most abundant [2,3]. The *COCH* gene is located on the long arm of chromosome 14 and encodes for the COCH protein, cochlin. This protein contains different domains: an N-terminal signal peptide (SP), an LCCL (Limulus factor C, cochlin, lung gestational protein) domain, two vWFA domains (von Willebrand factor A-like) and two short intervening domains (ivd) [4,5]. The exact function of cochlin is not fully understood but previous studies indicated that cochlin is involved in the clearance of bacterial infections in the inner ear where the LCCL domain is cleaved by aggrecanase-1 and secreted into the scala tympani [6]. The vWFA domains are believed to be involved in maintaining the structure of the extracellular matrix (ECM) due to their affinity for type I, type II and type IV collagens [5]. Cochlin is expressed in low levels in the vestibular labyrinth, spleen, lymph nodes, cerebellum and eye but it is abundantly expressed in the spiral ligament, spiral osseous, and spiral limbus of the inner ear [7].

Noise exposure can induce temporary (TTS) and permanent (PTS) threshold shifts resulting in noise-induced hearing loss (NIHL). NIHL recovers in 2–3 weeks, depending on initial severity, TTS will fully recover while PTS will stabilize at an elevated value [8]. Damage to sensory cells is irreversible because these cells are incapable of regeneration leading to cochlear dysfunction and permanent hearing loss [3]. The key mechanism in NIHL is the presence of oxidative stress in the cochlea involving the production of reactive oxygen species (ROS) and free radicals in cochlear tissues. In addition, cochlear inflammation is also a major contributor to noise-induced cochlear injury [9]. This inflammatory response involves a rapid recruitment and infiltration of inflammatory cells from the systemic circulation. There are various inflammation-related genes implicated in the cochlear inflammatory response, but the precise molecular pathways and mechanisms remain unknown [10]. Different mutations in the *COCH* gene can cause DFNA9. This is an autosomal dominant disorder characterized by progressive sensorineural hearing—and vestibular loss [2,4]. In contrast, DNFB110 is the autosomal recessive variant caused by inactivating variants that leads to congenital hearing loss that is not associated with vestibular dysfunction [11,12,13,14]. In order to gain a better understanding of the exact function of the COCH protein and get more insight in the pathology of these disorders, different mouse models were created: a mouse model that carries the G88E mutation in one and both alleles of the *Coch* gene (*Coch^G88E/G88E^* mice, *Coch^G88E/+^* mice) to study the pathology of DFNA9 and a mouse model that is knockout for the *Coch* gene (*Coch^−/−^* mice) to study the function of the COCH protein, representing recessive *COCH* (DFNB110) patients [15,16].

The objective of this study is to assess the long-term hearing and vestibular function of *Coch^−/−^* mice and to investigate the role of the COCH protein in inner ear inflammation after noise exposure. Hypothetically, as *COCH* maintains the ECM of the inner ear due to its affinity for other ECM proteins, we assumed that *Coch^−/−^* mice may suffer more from the NIHL due to alternations in their ECM. However, we brought forward an alternative hypothesis related to the role of *COCH* plays in the innate immune system: a decreased inflammatory response to noise exposure may potentially result in less hearing loss in the *Coch^−/−^* mice. The dual role of the COCH protein in ECM functioning and inner ear inflammation underscores the importance of this study, as well as the unpredictability of the outcome.

## 2. Results

### 2.1. Cochlin Deficiency Causes Hearing Impairment at the Highest Frequencies in Aged Mice

The COCH protein is abundantly expressed in the inner ear and plays a role in otovestibular functioning. Therefore, long-term follow up of hearing and vestibular function in *Coch^+/+^* and *Coch^−/−^* mice was assessed by Vestibular Dysfunction Rating (VDI), Forced Swimming Test (FST), Distortion Product Otoacoustic emissions (DPOAE) and Auditory Brainstem Response (ABR) measurements at 6 months, 12 months, 15 months and 24 months of age.

**Figure 1:**
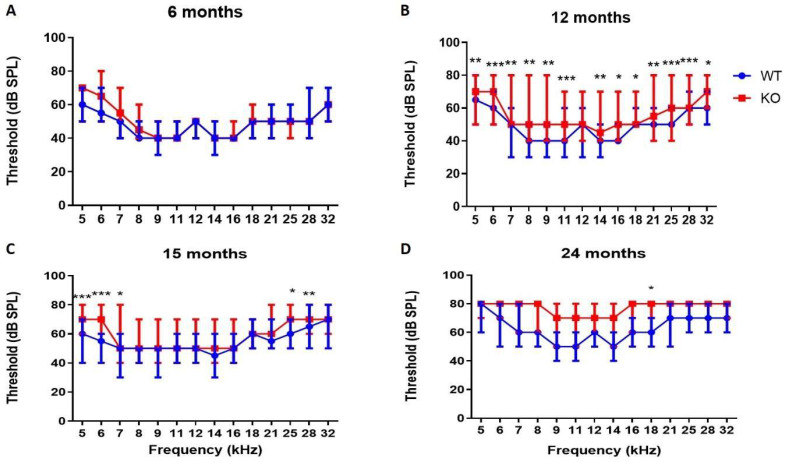
Distortion Product Otoacoustic Emissions (DPOAE) thresholds of *Coch^+/+^* (*n* = 12) and *Coch^−/−^* mice (*n* = 8) at 6 months (**A**), DPOAE thresholds of *Coch^+/+^* (*n* = 22) and *Coch^−/−^* mice (*n* = 30) at 12 months (**B**), DPOAE thresholds of *Coch^+/+^* (*n* = 10) and *Coch^−/−^* mice (*n* = 23) at 15 months (**C**) and DPOAE thresholds of *Coch^+/+^* (*n* = 5) and *Coch^−/−^* mice (*n* = 5) at 24 months (**D**). Data is represented as median with interquartile range. * indicates *p* < 0.05, ** indicates *p* < 0.01, *** indicates *p* < 0.001.

**Figure 2:**
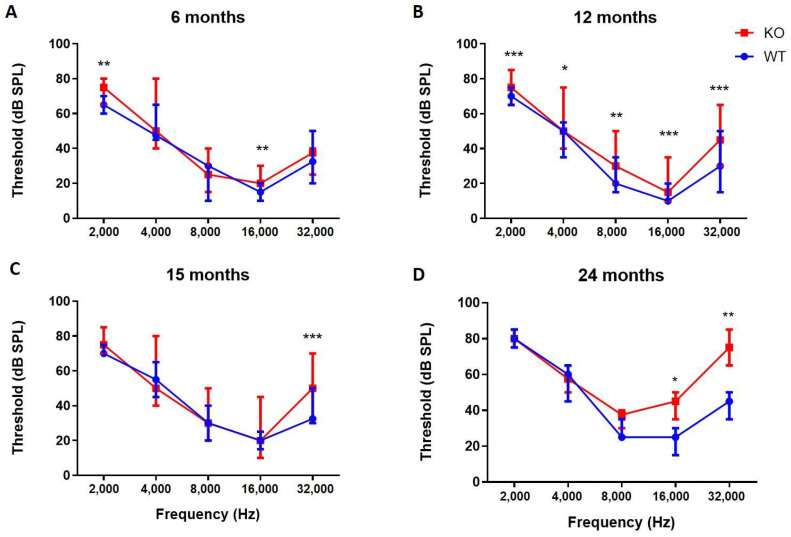
Auditory Brainstem Responses (ABR) thresholds between *Coch^+/+^* (*n* = 12) and *Coch^−/−^* mice (*n* = 8) at 6 months (**A**), ABR thresholds between *Coch^+/+^* (*n* = 22) and *Coch^−/−^* mice (*n* = 30) at 12 months (**B**), ABR thresholds between *Coch^+/+^* (*n* = 10) and *Coch^−/−^* mice (*n* = 23) at 15 months (**C**) and ABR thresholds between *Coch^+/+^* (*n* = 5) and *Coch^−/−^* mice (*n* = 5) at 24 months (**D**). Data is represented as median with interquartile range. * indicates *p* < 0.05, ** indicates *p* < 0.01, *** indicates *p* < 0.001.

- Vestibular evaluation. Similar to recessive *COCH* patients, normal vestibular function was observed in *Coch^−/−^* mice, even up to 24 months. Behavioral scorings of all mice in both groups remained within the normal control range (0 to 4) at all time points tested. No abnormal behavior was observed during the forced swimming test at the different time points in both groups.
- DPOAE. At 6 months of age, no differences were observed in DPOAE thresholds between *Coch^−/−^* (*n* = 8) and *Coch^+/+^* mice (*n* = 12) (Supplementary Table S1A) (Figure 1A). DPOAE measurements at 12 months of age demonstrate that thresholds of *Coch^−/−^* mice (*n* = 30) are significantly elevated compared to thresholds of *Coch^+/+^* mice (*n* = 22) at all frequencies except for 12 kHz (Supplementary Table S1A) (Figure 1B). However, at 15 months of age, DPOAE thresholds in *Coch^−/−^* mice (*n* = 23) are only significantly elevated at a few frequencies (5 kHz, 6 kHz, 7 kHz, 24 kHz and 28 kHz) compared to *Coch^+/+^* mice (*n* = 10) (Supplementary Table S1A) (Figure 1C). When mice reached the age of 2 years DPOAE measurements demonstrated no differences in thresholds between both groups (*n* = 5) except at 18 kHz (*p* = 0.017) (Supplementary Table S1A) (Figure 1D). These results indicate that *Coch^−/−^* mice develop temporary elevated DPOAE thresholds as they age but after two years, wildtype littermates had also elevated thresholds similar to *Coch^−/−^* mice.
- *ABR.* Long-term follow up of *Coch^−/−^* and *Coch^+/+^* mice to evaluate hearing function showed that *Coch^−/−^* mice had significant higher thresholds at 2 kHz and 16 kHz compared to *Coch^+/+^* mice as assessed by ABR recordings at the age of 6 months (Supplementary Table S1B) (Figure 2A). ABR measurements performed at the age of 12 months demonstrated that hearing thresholds in *Coch^−/−^* mice were significantly elevated compared to thresholds recorded in *Coch^+/+^* mice at all frequencies tested (Supplementary Table S1B) (Figure 2B). At the age of 15 months, ABR thresholds of *Coch^−/−^* mice were only significantly elevated at 32 kHz (Supplementary Table S1B) (Figure 2C) Hearing assessment at 24 months of age revealed that thresholds were significantly elevated at 16 and 32 kHz when compared to their wildtype littermates (Supplementary Table S1B) (Figure 2D). While recessive *COCH* patients suffer from congenital hearing loss, *Coch^−/−^* mice preserved their hearing function until the age of 2 years except for the high frequencies. The results of the ABR-measurements show increased thresholds in *Coch^−/−^* mice compared to wildtype, at some frequencies, depending on age. The difference seems most widespread at 12 months (all frequencies affected), while at 24 months only the high frequencies are affected.

### 2.2. Improved Hearing Recovery after Acoustic Overexposure with Cochlin Deficiency

Noise exposure can cause elevated hearing thresholds in mice. In order to investigate the effect of noise-exposure on hearing thresholds and vestibular function in *Coch^+/+^* mice (*n* = 13) and *Coch^−/−^* mice (*n* = 13), otovestibular functioning was assessed by performing VDR, FTS, DPOAE and ABR measurements before and after exposure to 120 dB broadband noise.

**Figure 3:**
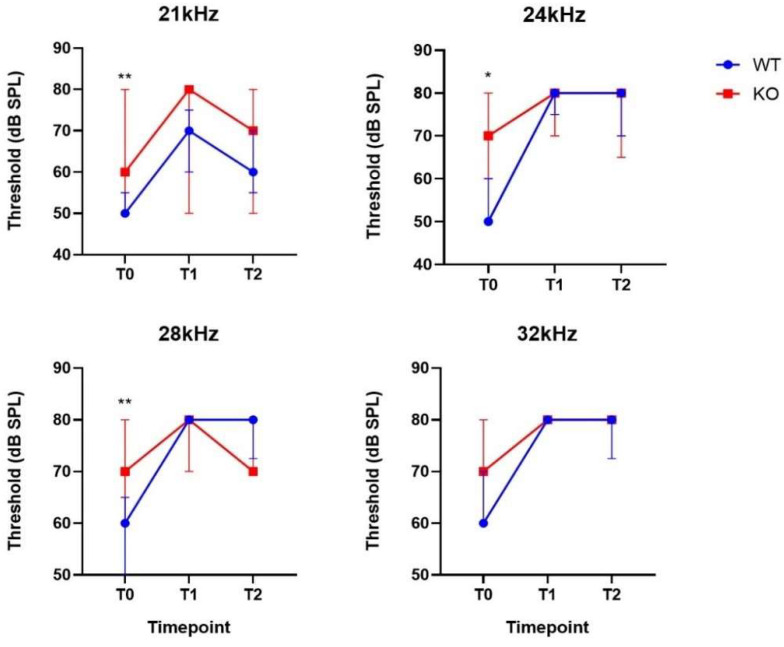
DPOAE thresholds at different time point (T0 = baseline, T1 = 48 h post-noise and T2 = one-week post-noise) between *Coch^+/+^* (*n* = 13) and *Coch^−/−^* mice (*n* = 13) that were exposed to noise at frequencies with a significant interaction between genotype and time. Data are represented as median with interquartile range. * indicates *p* < 0.05; ** indicates *p* < 0.01.

**Figure 4:**
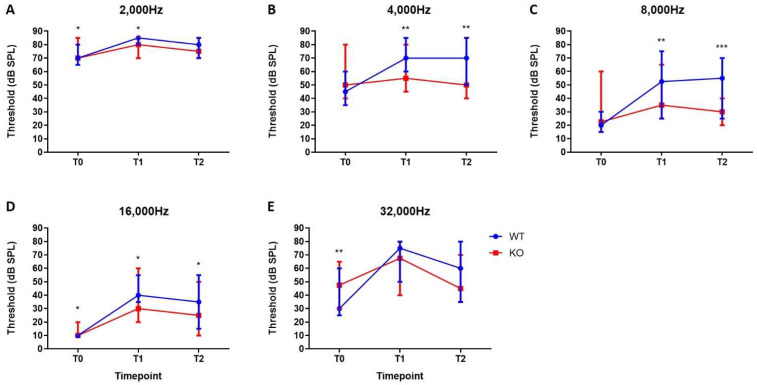
ABR thresholds of *Coch^−/−^* (*n* = 13) and *Coch^+/+^* mice (*n* = 13) at different timepoints (T0 = baseline, T1 = 48 h post-noise, T2 = one-week post-noise) at 2000 Hz (**A**), 4000 Hz (**B**), 8000 Hz (**C**), 16,000 Hz (**D**) and 32,000 Hz (**E**). Data are represented as median with interquartile range. * indicates *p* < 0.05, ** indicates *p* < 0.01, *** indicates *p* < 0.001.

- Vestibular evaluation. Vestibular rating scores remained within the normal control range (0 to 4) across all groups at all time point tested. Also, FST revealed no abnormal behavior in both *Coch^+/+^* and *Coch^−/−^* mice.
- *DPOAE.* Significant interactions between genotype and time were found in the noise exposure group at high frequencies (21–32 kHz) (Supplementary Table S2A). Post-hoc tests revealed that DPOAE thresholds were significantly elevated at 48 h post noise across all frequencies except from 6 kHz and 12k kHz in the *Coch^+/+^* mice. One week after noise trauma thresholds remained significantly elevated at all these frequencies except at 10.5 kHz and 14 kHz (Supplementary Table S2B). In the *Coch^−/−^* group, hearing thresholds were significantly elevated at all frequencies after 48 h except at 6 and 7 kHz but one week after noise trauma thresholds recovered to normal values in the mid-frequency region (10–21 kHz) (Supplementary Table S2B). These results demonstrate that hearing thresholds of *Coch^+/+^* did not recover following noise trauma while thresholds of *Coch^−/−^* mice recovered at all frequencies except for the low frequencies (5 kHz–9 kHz) and the high frequencies (24–32 kHz). Because of the early ARHL observed in *Coch^−/−^* mice, this group had significant higher thresholds than *Coch^+/+^* mice at baseline measurements at the high frequencies (21 kHz–28 kHz). Forty-eight hours and one week after noise exposure, there were no significant differences between both groups (Figure 3).

- *ABR.* A significant interaction between genotype and time was found in the noise exposure group revealing a different reaction between *Coch^−/−^* and *Coch^+/+^* mice following noise exposure at all frequencies (supplementary data, Table S3A). Post-hoc tests were performed to compare hearing thresholds at the different timepoints in both groups. Hearing thresholds of *Coch^+/+^* mice were significantly elevated at 48 h and one-week post-noise at all frequencies while hearing thresholds of *Coch^−/−^* mice were significantly elevated 48 h post noise at 2, 8, 16 and 32 kHz. In contrast to the *Coch^+/+^* mice, hearing thresholds of *Coch^−/−^* mice recovered to normal values after one week except at 16 kHz (supplementary data, Table S3B). A statistically significant difference was observed at 2 kHz at baseline where *Coch^−/−^* mice had higher ABR thresholds than *Coch^+/+^* mice (*p* = 0.018). In contrast, 48 h after noise exposure *Coch^−/−^* mice had significantly lower thresholds that *Coch^+/+^* mice (*p* = 0.04). One week after noise exposure there was no significant difference between both groups (Figure 4A). No difference in thresholds was observed at baseline at 4 kHz, whereas 48 h and one week after noise trauma significantly higher ABR thresholds were observed in the *Coch^+/+^* group compared to the *Coch^−/−^* group (*p* = 0.002 at 48 h and *p* = 0.006 at one week) (Figure 4B). At 8 kHz, baseline measurements revealed no difference in hearing thresholds between both group but *Coch^+/+^* mice had significant higher thresholds after noise exposure at 48 h (*p* = 0.004) and one-week post-noise (*p* = 0.0001) (Figure 4C). Baseline measurements at 16 kHz demonstrate that *Coch^−/−^* mice had significant higher thresholds than *Coch^+/+^* mice (*p* = 0.015) while after noise exposure *Coch^+/+^* mice had significant higher thresholds that *Coch^−/−^* mice (*p* = 0.01 at 48 h and *p* = 0.03 at one week) (Figure 4D). Results at 32 kHz showed a significant difference in thresholds at baseline where *Coch^−/−^* mice had significant higher thresholds than *Coch^+/+^* mice (*p* = 0.0047), after noise exposure, no differences in hearing thresholds were observed between both groups (Figure 4E). Hearing assessment indicate that *Coch^+/+^* mice are more affected by noise exposure than *Coch^−/−^* mice as their hearing thresholds did not recover one week after noise exposure in contrast to the hearing thresholds of their *Coch* knockout littermates.

**Figure 5:**
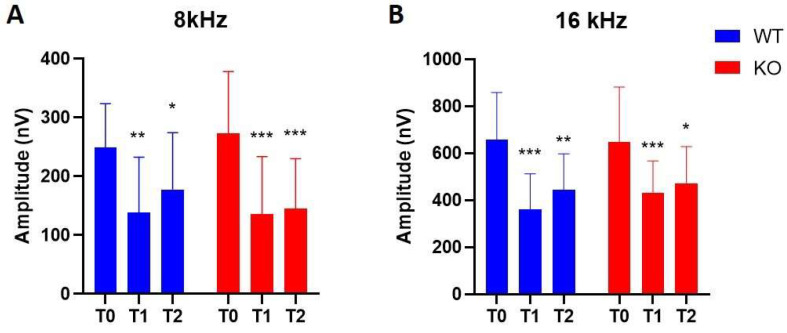
Wave I amplitudes of *Coch^−/−^* (*n* = 13) and *Coch^+/+^* mice (*n* = 13) at different timepoints (T0 = baseline, T1 = 48 h post-noise, T2 = one-week post-noise) at 8 kHz (**A**), and 16 kHz (**B**). Data are represented as mean with SD. * indicates *p* < 0.05, ** indicates *p* < 0.01, *** indicates *p* < 0.001.

Linear mixed models followed by post hoc tests revealed a significant decrease in wave I amplitudes 48 h after noise exposure in both groups at 8 kHz and 16 kHz when compared to baseline measurements. Wave I amplitudes remained significantly decreased after one week of noise exposure in both *Coch^+/+^* and *Coch ^−/−^* mice (Supplementary Data Table S4A,B) (Figure 5). Direct comparison by Mann–Whitney U tests of wave I amplitude between *Coch^+/+^* and *Coch ^−/−^* mice revealed no statistical differences between both groups at 8000 Hz (*p* = 0.54 at baseline, *p* = 0.73 48 h post noise and *p* = 0.98 one week post noise). Also, at 16 kHz, no statistical differences were found in wave I amplitudes between both groups at baseline (*p* = 0.99), 48 h after noise (*p* = 0.61) and one week after noise exposure (*p* = 0.28). 

### 2.3. Immunohistochemistry

One week after noise exposure, mice belonging to the noise-exposure groups and control groups were euthanized. One cochlea was used to perform whole mount dissection of the organ of Corti followed by staining with an anti-myosin VIIa antibody and an anti-synaptophysin antibody to visualize hair cells and neurons, respectively. The other cochlea was used to make cryosections of the spiral ligament in order to assess fibrocyte integrity, inner ear inflammation and Cochlin expression.

### 2.4. Hair Cells and Neurons Remained Intact after Noise Trauma

**Figure 6:**
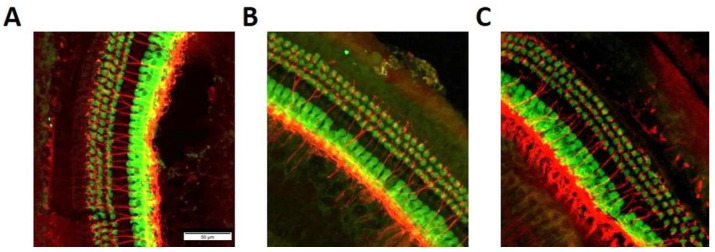
Whole mount staining of the organ of Corti in the basal turn after noise exposure of a wildtype mouse (**A**) a *Coch^−/−^* mouse (**B**) and a control mouse that was not exposed to noise (**C**). No structural damage of hair cells (anti-Myosin VIIa, green) or neurons and synapses (anti-synaptophysin, red) was observed in both groups.

Whole mount dissection and staining of the organ of Corti revealed that hair cells and neurons remained intact after noise exposure. Hair cell counts using ImageJ revealed that no statistical difference was observed in IHC and OHC across all groups (*p* = 0.16 and *p* = 0.69, respectively). (Figure 6).

### 2.5. Noise Exposure Did Not Cause an Inflammatory Reaction in the Spiral Ligament or Spiral Limbus

**Figure 7:**
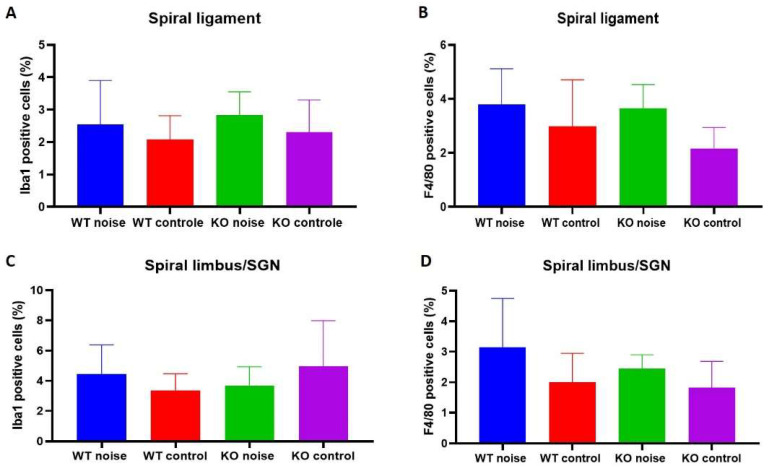
IBA1 staining in the spiral ligament (**A**) and the spiral limbus/spiral ganglion neuron region (**C**) across all groups. F4/80 staining in the spiral ligament (**B**) and spiral limbus/spiral ganglion neuron region (**D**) show no significant difference in F4/80 positive cells between *Coch^+/+^* mice exposed to noise (*n* = 13), *Coch^+/+^* control mice (*n* = 9), *Coch^−/−^* mice exposed to noise (*n* = 13) and *Coch^−/−^* control mice (*n* = 13). Positive cells were counted using ImageJ and divided by the total amount of cells to calculate a percentage.

To assess inner ear inflammation, staining with IBA1 and F4/80 antibodies in the spiral ligament and spiral limbus/spiral ganglion neuron region was performed to visualize macrophages and activated macrophages, respectively. No significant difference in macrophage influx in the spiral ligament or the spiral limbus was observed across all groups (Figure 7).

### 2.6. Spiral Ligament Fibrocytes Were Not Affected by Noise Trauma in Coch^−/−^ and Coch^+/+^ Mice

**Figure 8:**
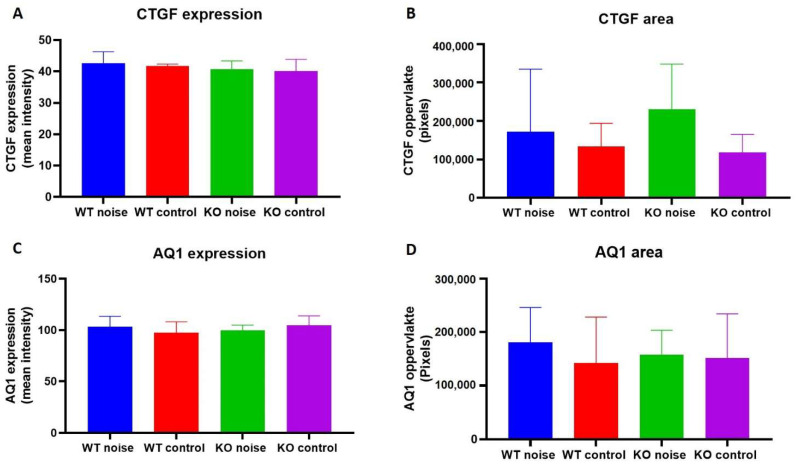
CTGF expression (**A**) and area of expression (**B**) in the spiral ligament across all groups. AQ1 expression (**C**) and area of expression (**D**) in the spiral ligament between *Coch^+/+^* mice exposed to noise (*n* = 13), *Coch^+/+^* control mice (*n* = 9), *Coch^−/−^* mice exposed to noise (*n* = 13) and *Coch^−/−^* control mice (*n* = 13). No significant differences were found in expression level and expression area of type III and type IV fibrocytes.

*Coch* expression is most abundant in the region where the type IV fibrocytes are located [2]. As these cells are suggested to be highly vulnerable to noise trauma, staining with an anti-CTGF antibody was performed in order to visualize type IV fibrocytes. No significant differences could be observed in expression level of these fibrocytes across all groups (Figure 8A) as well as the area of expression (Figure 8B). Type III fibrocytes are known for their self-renewal capacities and exhibit stem cell abilities. Staining with an anti-AQ1 antibody revealed no differences in expression level (Figure 8C) and area of expression (Figure 8D) of type III fibrocytes among all groups. Also, no migration of type III fibrocytes to other regions in the spiral ligament could be observed.

### 2.7. Noise Trauma Reduces Cochlin Immunoreactivity in the Spiral Ligament

**Figure 9:**
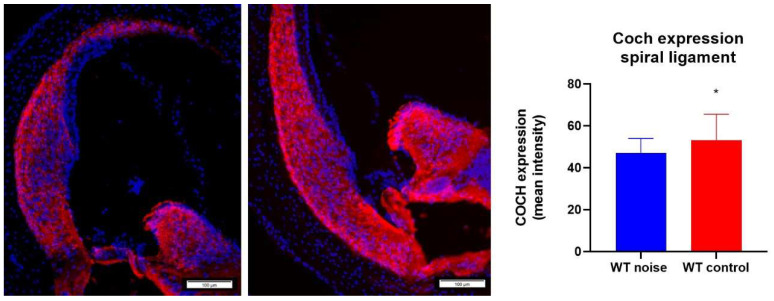
Cochlin expression (*COCH* antibody, red) in the spiral ligament between *Coch^+/+^* mice that were exposed to noise (n = 13) (**left**) and *Coch^+/+^* mice that belong to the control group (*n* = 9) (**middle**). *Coch* expression was measured in ImageJ. A significant difference was found in *Coch* expression between both group where the noise exposure group had significant lower levels of *Coch* expression than the wildtype control group. * indicates *p* < 0.05.

An anti-*Coch* antibody was used to stain for the COCH protein in the spiral ligament. It was observed that *Coch^+/+^* mice that were exposed to noise had a significant lower expression level of the COCH protein than *Coch^+/+^* mice that were not exposed to noise (Figure 9).

## 3. Discussion

### 3.1. Otovestibular Functioning of the Coch Knockout Mouse Model

Our study revealed that ABR thresholds of *Coch^−/−^* mice were significantly elevated compared to ABR thresholds of *Coch^+/+^* mice at the age of 12 months at all frequencies. After two years of age, ABR thresholds of *Coch^−/−^* mice remained only elevated at 16 kHz and 32 kHz. A similar pattern was seen in the analysis of the DPOAE measurements after one year where thresholds were also significantly elevated in the *Coch^−/−^* mice at all frequencies, except 12 kHz. After two years only the DPOAE thresholds measured at 18 kHz remained significantly elevated in the *Coch^−/−^* mice compared to the *Coch^+/+^* mice. We believe that the significant difference in hearing thresholds observed after one year is due to the large sample size of *Coch^+/+^* (*n* = 22) and *Coch^−/−^* mice (*n* = 30). After this timepoint, mice were included in the noise exposure groups and died from natural causes resulting in a decreased sample size for the measurements at 15 months and 24 months. Also, hearing function of *Coch^+/+^* mice deteriorated at older ages compensating for the hearing loss observed in *Coch^−/−^* mice in the first 12 months of age.

Similarly, another study found that *Coch^−/−^* mice had significant elevated hearing thresholds when compared to their wildtype littermates at high frequencies and hearing thresholds were completely absent at 41.2 kHz at the age of 21 months [16]. 

In contrast to our study, Jones et al. found vestibular dysfunction starting from the age of nine months, while in our study, no vestibular dysfunction was observed. It is important to note that we used the VDI which is based on observation of reflexes and behavior while Jones et al. used vestibular sensory-evoked potential (VSeP) measurements [16]. VSeP recordings are carried out by using subcutaneous electrodes placed over the nuchal crest, left pinna and the hip. The head of the mice is secured by a noninvasive head clip to a mechanical shaker for the delivery of linear vestibular stimuli [16]. VeSP is an electrical potential that provides a direct test of vestibular function in animals while VDI is based on observation and behavior of the animals and, therefore, a more subjective test. 

According to our study *Coch^−/−^* mice did not exhibit vestibular dysfunction, which is similar to the phenotype observed in recessive *COCH* patients. In contrast, *Coch^−/−^* mice have higher ABR and DPOAE thresholds compared to *Coch^+/+^* mice at the age of one year while recessive *COCH* patients suffer from congenital hearing loss [11,12].

### 3.2. Hearing Function after Noise Trauma

Previous studies have demonstrated that cochlin plays a role in coordinating immune responses elicited by bacterial infection of the cochlea [6,17]. As *Coch^−/−^* mice lack the *Coch* gene, we would expect the inflammatory response to be less pronounced in *Coch^−/−^* mice. A decreased immune response could result in less permanent hearing loss as inflammation can further damage the inner ear. On the other hand, the immune response helps clear the debris of damaged cells in the cochlea and can therefore prove to be beneficial to the hearing function [3].

Our results show that there was a significant difference in DPOAE thresholds after noise exposure where thresholds of *Coch^−/−^* mice recovered to baseline values one week after noise exposure in the mid frequency region of 10 kHz to 21 kHz while DPOAE thresholds of *Coch^+/+^* mice remained elevated at all frequencies. A similar observation can be drawn from ABR measurements where thresholds of *Coch^−/−^* mice recovered to baseline values one week after noise exposure except at 16 kHz while ABR thresholds of *Coch^+/+^* mice remained elevated at all frequencies. Similar to our study, Seist et al. discovered that *Coch^−/−^* mice had significantly lower DPOAE-and ABR thresholds shifts when compared to *Coch^+/+^* mice following exposure to 103 dB of broadband noise (8–16 kHz) for 2 h [18]. Threshold shifts recovered after one week are considered as TTS. Elevated thresholds remaining after one week could be either temporary or permanent as TTS can persist up to two weeks [19]. Threshold shifts are usually caused by damage to both sensory and non-sensory cells in the inner ear [3]. Whole mount dissection was performed to assess hair cell and neural integrity. Staining revealed that there was no loss of hair cells and neurons in both groups indicating another mechanism is responsible for the difference in response to noise exposure that is observed between *Coch^+/+^* and *Coch^−/−^* mice. In contrast, Seist et al. observed OHC loss in both *Coch^−/−^* and *Coch^+/+^* mice after noise exposure where the number of surviving OHC was significantly higher in *Coch^−/−^* mice when compared to wildtype animals [18]. It is important to note that the mice that were used in their study were only 6 weeks old and NIHL vulnerability varies with age at exposure. Kujawa and Liberman discovered that young mice are more susceptible to noise than older mice. Moreover, the age of 4–6 weeks is a critical period for noise vulnerability in mice [20].

A reduction in wave I amplitudes at 8 kHz and 16 kHz was observed in both groups following noise-exposure. Decreased wave I amplitudes could indicate a reduction in auditory nerve fibres due to noise-exposure, this phenomenon is called synaptopathy [21]. Noise exposure may cause significant loss of ribbon synapses while no apparent loss of IHCs is observed [22].

### 3.3. Inner Ear Inflammation Following Noise Exposure

Although the role of cochlin is not yet fully understood, the LCCL domain of cochlin is involved in promoting immune responses [17]. The LCCL domain has strong homology with Factor C, an endotoxin-sensitive serine proteinase involved in the immune response in the horseshoe crab Limulus where it functions as an antibacterial peptide [6,23]. In addition, cochlin has been identified as an important modulator of immune responses in the spleen and lymph nodes where it is involved in the regulation of macrophage activation, recruitment of immune cells and cytokine production [17]. Furthermore, it has been previously reported that noise exposure leads to inflammation in the inner ear with an influx of macrophages, specifically into the spiral ligament [24]. 

A recent study [6] demonstrated that *Coch^−/−^* mice have a significantly decreased immune response after a bacterial infection in the inner ear as functional cochlin is necessary to induce an innate immune response. Basal populations of resident immune cells were similar between wildtype mice and *Coch^−/−^* mice in the cochlea. However, after infection, the number of infiltrated neutrophils, macrophages, and dendritic cells was significantly lower in *Coch^−/−^* mice. Baseline cytokine expression did also not differ between wildtype and *Coch^−/−^* mice. However, post infection, levels of IL-1β and IL-6 were significantly elevated in *Coch^+/+^* mice compared to *Coch^−/−^* mice, suggesting that cochlin is necessary for local upregulation and increased secretion [6]. Another study [25] observed a massive neutrophil migration in the spiral ligament two days after LPS inoculation in the middle ear. In contrast, noise exposure did not induce migration of neutrophils in the lateral wall. Noise exposure and bacterial infection with LPS have different inflammatory pathways because LPS is recognized as a pathogen-associated molecular pattern, but debris from the degenerated cells after noise exposure is recognized as a damage-associated molecular pattern (DAMP) [25].

Interestingly, our results suggest that *Coch^−/−^* mice suffer less from noise exposure compared to wildtype mice but no differences in IBA1 and F4/80 positive macrophages were found in the spiral ligament and spiral limbus region between both groups. Seist et al. observed an increased expression of the Adamts4 gene which is responsible for the cleavage of the LCCL domain of cochlin initiating immune response. In addition, the expression of genes encoding for the proinflammatory cytokines IL6 and CXCL1 was upregulated in the perilymph of *Coch^+/+^* mice 6 h post noise exposure when compared to *Coch^−/−^* mice [18]. These results suggest that cochlin may be involved in the immune response after noise exposure in young mice, but this upregulation does not lead to an increase of macrophages in the inner ear of older mice as indicated by our findings. Further research regarding cytokine expression and influx of other immune cells in the inner ear after noise exposure in adult *Coch^+/+^* and *Coch^−/−^* mice should be performed in order to confirm our findings. 

### 3.4. Fibrocyte Integrity

The spiral ligament consists of five different fibrocyte types, all with distinct functions. Type I spiral ligament fibrocytes (SLFs) are located adjacent to the stria vascularis where they are associated with collagen bundles, type II SLFs play an important role in potassium recycling and are located near the spiral prominence. These two SLF types make up most of the spiral ligament. The bony otic capsule is confined by small elongated type III SLFs, the spindle-shaped type IV SLF are located inferiorly, towards the crista basilaris, while type V SLF are located near the apical tip, where they make direct contact with the perilymph of the scala vestibuli [2,26,27,28]. Fibrocytes in the spiral limbus and spiral ligament play a role in maintaining the endocochlear potential in the scala media by recycling of K^+^ ions. Loss of fibrocytes disrupts the K^+^ recycling causing an abnormal K^+^ concentration in the endolymph which influences the normal activation of the hair cells resulting in hearing loss [2,29,30].

In previous studies, 94 dB was considered as the border for irreversible damage and the threshold for degeneration of type IV SLFs. We stained with an anti-CTGF antibody which is highly selective for type IV SLFs [31]. No significant differences were observed in expression and area of type IV SLFs, suggesting no degeneration of these fibrocytes occurred after noise trauma. This is in contrast with other studies reporting that loss of type IV SLFs can be observed at all levels of noise exposure starting from 94 dB [2,31]. Similar to our study, Cui et al. [29] exposed mice to 120 dB noise and did not detect significant fibrocyte loss in the spiral ligament up to 8 weeks following noise exposure. The discordance between the studies is most probably due to the genetic background of the mice used in these studies as it was previously showed that different strains of mice demonstrate significant heterogeneity in noise susceptibility [32,33].

An interesting observation is that type III SLFs do not degenerate significantly after noise exposure. In fact, the number of proliferating cells increase in the type III SLF region. These fibrocytes can repopulate the type I fibrocyte region after loss of these cells. Type III SFLs express AQ1 which is a protein involved in cell migratory mechanisms giving them the possibly to migrate through the spiral ligament. Furthermore, type III SFLs are considered to have stem cell abilities. Therefore, type III SLFs could play a potential role in regenerative therapies [26,34,35]. 

Type III SFLs were visualized using an AQ1-antibody but no differences were found in AQ1-expression and area across all groups. This finding is consistent with the observation that there was no damage to the type IV SFLs which are most sensitive to noise exposure. As no fibrocyte loss has occurred in the spiral ligament, there was no need for the type III SFLs to upregulate and migrate through the spiral ligament to repopulate areas of fibrocyte loss. 

### 3.5. Coch Expression 

To assess whether cochlin could be responsible for the difference in hearing thresholds that was observed between *Coch^+/+^* and *Coch^−/−^* mice after noise exposure, *Coch* expression in wildtype mice was assessed using an anti*-COCH* antibody. We found that *Coch* expression was significantly decreased in *Coch^+/+^* mice after noise exposure, which could be explained by the observation of Seist et al. that cleaved cochlin is upregulated in *Coch^+/+^* mice following noise exposure [18].

### 3.6. Alteration in ECM Proteins May Contribute to Hearing Loss after Noise Exposure

Seist et al. concluded that the difference in threshold shifts after noise exposure in *Coch^−/−^* mice is due to a small conductive hearing loss observed at the age of 6 weeks in these mice attenuating the amount of noise carried in the cochlea and thereby preventing some of the noise-induced damage [18]. In our study, we followed up *Coch^+/+^* and *Coch^−/−^* mice until the age of two years to assess hearing function with six months being the first timepoint where measurements were performed. At this time, we only found differences in ABR thresholds at 2 kHz and 16 kHz and no differences were found across all DPOAE frequencies between *Coch^−/−^* and *Coch^+/+^* mice. Even at the age of 24 months, hearing thresholds in *Coch^−/−^* mice were only elevated at the highest frequencies compared to wildtype mice. Based upon our observations, we believe there should be another reason responsible for the different responses in *Coch^+/+^* and *Coch^−/−^* mice following noise-exposure that are outlined below. 

Besides a role in the clearance of bacterial infections in the inner ear, cochlin has a function in maintaining the structure of the ECM of the inner ear by the affinity of the vWFA domains for type I, type II and type IV collagens [5]. The vWFA2 domains of cochlin have a structure similar to other vWFA domains: a central beta-sheet of six strands, flanked by three and four helices. It has a metal ion-dependent adhesion site (MIDAS) motif, which plays an important role in structural stability and ligand binding in vWFA domain-containing proteins [36].

A downregulation of *Coch* following noise exposure may cause alterations in the ECM proteins which may lead to a disruption of homeostasis in the inner ear causing hearing loss [37]. A similar phenomenon has been observed in the eye, where upregulation of cochlin results in a higher intraocular pressure (IOP) causing glaucoma [38,39]. Goel et al. [37]. observed that when cochlin was downregulated by injection of *Coch*-shRNA, the IOP decreased and the decreased IOP level was maintained for the next two months. The vWFA domain present in ECM proteins is associated with fluid shear responsiveness indicating that cochlin may potentially act as a mechanosensing molecule [37]. Increased fluid shear leads to cochlin multimerization, which is resistant to proteolysis and can potentially accumulate in the ECM of the trabecular meshwork in glaucomatous eyes [38]. As both the anterior eye chamber and the perilymph space of the ear contain fluid, it is speculated that cochlin may have a function in maintaining the shear stress and ion homeostasis of these fluids by interaction with collagen II to build up the ECM. [5,37,39].

## 4. Materials and Methods

### 4.1. Study Design

**Figure 10:**
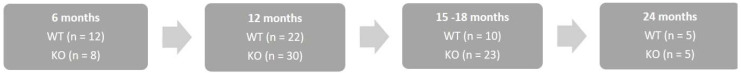
Study design of the long-term follow up of *Coch^+/+^* and *Coch^−/−^* mice.

*Coch^+/+^* CBACa.129S1(Cg)-Cochtm1.1Stw/Mmjax (*Coch* wildtype) and *Coch^−/−^* CBACa.129S1(Cg)-Cochtm1.1Stw/Mmjax (*Coch* knockout) were followed up for a period of two years for otovestibular functioning. Otovestibular testing was performed at 6 months, 12 months, 15–18 months, and 24 months. The outline of this study design can be seen in Figure 10.

To investigate the effect of noise exposure on hearing thresholds both adult mice (age between 7–9 months) and old mice (age between 14–16 months) were included. At the start of the experiment mice were randomly allocated to a noise exposure group and a control group. The noise exposure group included adult *Coch^+/+^* mice (*n* = 7), old *Coch^+/+^* mice (*n* = 6), adult *Coch^−/−^* mice (*n* = 7) and old *Coch^−/−^* mice (*n* = 6). The control groups consisted of adult *Coch^+/+^* mice (*n* = 7), old *Coch^+/+^* mice (*n* = 2), adult *Coch^−/−^* mice (*n* = 6), and old *Coch^−/−^* mice (*n* = 7). 

**Figure 11:**
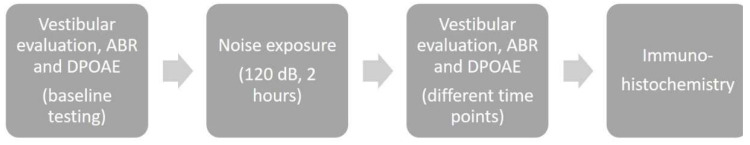
Schematic study design.

First, baseline testing was performed in all mice (*n* = 48) to assess the vestibular and hearing function before noise exposure. After baseline testing, the noise exposure group was exposed to 120 dB broadband noise for 2 h. Vestibular and auditory testing was performed at two different time points (48 h and 1 week) after noise exposure. At one-week post-exposure, the mice were euthanized using pentobarbital (200 mg/mL) for immunohistochemical research. A schematic overview of the study design can be seen in Figure 11.

### 4.2. Animals

*Coch^+/−^* CBACa.129S1(Cg)-Cochtm1.1Stw/Mmjax mice were obtained from The Jackson laboratory (034310-JAX) and further bred at the University of Antwerp. All animal experimental procedures were approved by the Ethics Committee for Animal Experiments of the University of Antwerp (approval No 2019-46). Mice were housed four-five per cage in standard type III plastic cages with wood shavings as bedding and given water and standard pelleted rodent chow ad libitum. Cages were stored in sound-proof rooms at constant room temperature (20–24 °Celsius) and humidity (45%). They were maintained on a 12 h/12 h light–dark cycle. 

### 4.3. Noise Exposure

Awake mice were placed in a subdivided cage inside a soundproof box. The noise signal was generated using RPvdsEX software (Tucker-Davis technologies, Alachua, FL, USA). The signal was routed through an attenuator and power amplifier (XPS-1200, Gemsound, New York, NY, USA) to a high-frequency tweeter speaker (HTH 8.7, Visaton, Haan, Germany) positioned immediately above the mice holding cage. Broadband noise (8–16 kHz) of 120 dB was generated during a time period of 2 h. Mice were monitored the whole duration of the exposure via a webcam.

### 4.4. Vestibular Evaluation

#### 4.4.1. Vestibular Dysfunction Rating

To evaluate the vestibular function, the VDR was used. This is a validated in vivo score that correlates with bilateral vestibular failure. The test battery consisted of several criteria, both spontaneous motor behavior and vestibular reflexes that were scored from 0 (normal behavior) to 4 (extreme change in behavior). A description of the rating scores is provided in Table 1. First, the mice were placed in an open cage for 1 min to assess spontaneous motor behavior (circling, retropulsion and head bobbing). Circling was defined as stereotypical circulation, retropulsion showed a persistent backward movement and head bobbing consisted of intermittent extreme backward extension of the neck. Next, the mice were rated for different vestibular reflexes (tail lift reflex, contact inhibition of righting reflex, and air-righting reflex). The tail lift reflex consisted of a spreading of the forepaws when being picked up by the tail as a “landing” response. Mice with a weakened or impaired vestibular function curl their body ventrally, crawling up towards their tail. For the contact inhibition of the righting reflex, mice were in a supine position on a table and a plastic board was placed in contact with the mice. Healthy, normal mice will right themselves quickly to a normal position. Vestibular deficient mice, however, remain lying on their back with their feet up. For the air-righting reflex, mice were dropped supine from a height onto a foam cushion. Normal mice right themselves in the air during the fall, whereas vestibular deficient mice do not and land on their back or side. 

#### 4.4.2. Forced Swimming Test 

In addition to VDR, a FST was performed. Mice were placed in a transparent cage filled with 15 cm water of 25 °C. The swimming behavior of the mice was evaluated by two supervisors during 15 s according to the following score system: normal swimming (score 0), circling (score 1), tumbling or going beneath the surface (score 2).

### 4.5. Hearing Evaluation

#### 4.5.1. Anaesthesia

ABR and DPOAE measurements were performed under ketamine/xylazine anaesthesia. DPOAE measurements were done before ABR assessment to avoid reduced DPOAE responses, measurements were conducted consecutively without awakening of the animal. Mice were anaesthetized with an intraperitoneal (i.p.) injection with a ketamine (100 mg/kg body weight) and xylazine (20 mg/kg body weight) mixture. Reflexes were assessed by a hind limb withdrawal reflex, in case mice did not reach an areflexive state, boosters of ketamine/xylazine one-fifth of the original dose were administered fifteen minutes after anaesthesia induction. Hearing assessment was started fifteen minutes after ketamine/xylazine injection. 

After anaesthesia induction, mice were individually placed in a sound-attenuating chamber (Industrial Acoustic Company, North Aurora, IL, USA) on a homeothermic heating pad system (Harvard Apparatus, Holliston, MA, USA) to maintain constant body temperature (37 ± 0.5 °C). Prior to each recording session, ophthalmic ointment (Duratears, Alcon) was applied to the eyes to prevent corneal drying.

#### 4.5.2. Distortion Product Otoacoustic Emissions (DPOAE)

Anaesthetized mice were placed on their left flank under a device securely holding an acoustic probe tightly fitted into the right external auditory canal. Two tones (f1 and f2) were administered simultaneously in the right ear only via a close-field method. DPOAE responses (2f1−f2) were measured over a frequency range from 5 to 32 kHz, more specifically at 5.3, 6.1, 7.0, 8.0, 9.2, 10.6, 12.1, 13.9, 16.0, 18.4, 21.1, 24.3, 27.9, and 32.0 kHz. The primary tone ratio f2/f1 was set to 1.22. DPOAE responses were evoked by a non-symmetric DPOAE protocol, using unequal primary tone stimulus intensities (i.e., L1 > L2). Five intensity levels were presented with L1 going from 70 to 30 dB SPL and L2 = L1 − 10 dB SPL. Duration of testing was approximately 20 min per animal. To enable statistical analysis and calculations of the mean, unobtainable DPOAE thresholds at our equipment’s limits of 70 dB SPL were defined as 80 dB SPL.

#### 4.5.3. Auditory Brainstem Responses (ABR)

ABR thresholds were determined using procedures described previously, with minor modifications [40]. Evoked responses were recorded using disposable subcutaneous needle electrodes (28 G) positioned over the vertex of the skull (active electrode), the left mastoid (reference electrode) and the right hindlimb (ground electrode). Electrode placement was manipulated until an impedance of no higher than 2 kOhm was observed. Evoked potentials were measured after administration of frequency-specific sound stimuli through a free-field electrostatic speaker placed 10 cm in front of the animal’s head. BioSig32 software (Tucker-Davis Technologies, Alachua, FL, USA) was used to generate tone burst stimuli of 2 msec in length with a gate of 1 msec at frequencies 2, 4, 8, 16 and 32 kHz in 5 dB steps starting at 80 dB SPL down to a minimum SPL of 10 dB. A stimulus repetition rate of 32 per second was used and 800 trials were recorded for each frequency to obtain a good averaged response. Since potentials have very weak amplitude, averaging of 800 stimulations was essential to differentiate from background noise. ABR thresholds were defined as the lowest stimulus level at which any reproducible ABR waveform could be reliably observed in the evoked response at appropriate latencies upon visual inspection and was determined by comparing the ABR waveforms with several suprathreshold ABRs. Threshold analyses were performed via offline analysis of stored waveforms, and the thresholds obtained for each frequency were verified at least twice by the same experimenter. In addition, amplitudes of wave I were assessed at 8 and 16 kHz. At the other frequencies, this parameter was not analyzed because it was difficult to identify wave I at 2, 4 and 32 kHz. Upon completion of testing, needle electrodes were removed, and animals were moved individually to heated cages and monitored until complete recovery. Duration of testing was approximately 30 min per animal. To enable statistical analysis and calculations of the mean, unobtainable ABR thresholds at our equipment’s limits of 80 dB SPL were defined as 85 dB SPL.

### 4.6. Euthanasia

Mice were euthanized with an overdose of pentobarbital (200 mg/mL). 

### 4.7. Immunohistochemistry

The skull of the mouse was cut open with scissors and brain tissue was removed to expose the temporal bones bilaterally. The otic capsule and surrounding tissue was removed so a small incision can be made in the apex. The cochleae were fixated in 4% paraformaldehyde (PFA) for 1 h. Afterwards, they were washed 3 × 5 min with PBS and the cochleae were put in 0.5 M EDTA overnight. Next, the cochleae were either used for whole mount dissection or cryosection.

#### 4.7.1. Whole Mount Dissection of the Mouse Cochlea

The cochleae were washed with PBS and the organ of Corti was dissected. The apex, middle turn and basal turn were put in 2 mL Eppendorf filled with PBS and later placed in a 24-well plate for staining. To permeabilize the tissues, they were placed in Triton-x-100 4% and later blocked with Triton-x-0.5% and Fish gelatin 1%, both for 1 h at room temperature. The tissues were incubated overnight with primary antibodies: Rabbit anti-myosin VIIa (Proteus Biosciences (25-6790), 1:200 dilution in Triton-x-0.1% and Fish gelatine 1%) and Guinee Pig anti-synaptophysin (synaptic systems (101004), 1:2000) to stain hair cells and spiral ganglion neurons, respectively. Next, samples were washed 3 × 5 min with PBS and incubated with secondary antibodies Goat anti-rabbit Fluor 555 (Abcam (ab6719), 1:1000 dilution in Triton-x-0.1% and Fish gelatine 1%) and Goat anti-mouse FITC (Dako (F0313), 1:1000) for 1 h at room temperature and washed again 3 × 5 min with PBS. At last, the samples were incubated with DAPI (1:1000) at room temperature for 10 min and cleaned 3 × 5 min with PBS. Samples were visualized using Leica SP 8 laser scanning confocal microscopy (Leica Microsystems, Mannheim, Germany). ImageJ was used for image acquisition and processing.

#### 4.7.2. Cryosections and Immunohistochemical Staining

The cochleae were washed with PBS and put in different sucrose concentrations (2 h in sucrose 5%, 2 h in sucrose 10% and overnight in sucrose 20%). Next, cryosectioning was performed in the desired orientation and the slices were washed with PBS for 3 × 5 min and placed in 0.1% Triton 10× for 30 min and later put in blocking solution (20% goat serum and 20% donkey serum in PBS) for 1 h. The slices were incubated overnight with primary antibodies at 4 °C, next slices were washed 4 × 3 min with PBS. Next, secondary antibodies were added and incubated for 1 h at room temperature. Then the slices were washed with PBS and DAPI (1:1000) was added for 10 min. Afterwards, the samples were washed 2 × 2 min with demi water and mounted with 1 drop of ProGold. Slices were dried and visualized using Olympus BX51 fluorescence microscope equipped with an Olympus DP71 digital camera. Olympus CellSens software was used for image acquisition and processing. ImageJ was used to quantify macrophages and measure CTGF and *Coch* expression using two sliced per cochlea for each mouse. An overview of the antibodies that were used for this IHC staining is given below in Table 2.

### 4.8. Statistical Analysis

Mann–Whitney U tests were performed to analyze hearing thresholds between *Coch^+/+^* and *Coch^−/−^* mice at the different time points tested during the long-term follow up.

To assess the effect of noise exposure on hearing functioning in mice, linear mixed models were fitted to analyze the interactions between time, genotype and age. Also, LLM’s were fitted to explore the main effects of time*KO and time*WT. Post-hoc tests were performed when main effects were significant. To analyze the difference in hearing thresholds between *Coch^−/−^* and *Coch^+/+^* mice at the two timepoints after noise exposure Mann–Whitney-U tests were performed. IHC stainings were analyzed using ImageJ to count immunoreactive cells and assess expression level of the staining.

Linear mixed models revealed a significant interaction between genotype and time in the noise exposure group, but no significant interaction was observed between age and time or age and genotype. Therefore, adult and old mice were taken together as one group for the analysis of vestibular function, hearing assessment and immunohistochemistry. Statistical analysis was performed using 4 different groups: *Coch^+/+^* noise (*n* = 13), *Coch^−/−^* noise (*n* = 13), *Coch^+/+^* control (*n* = 9) and *Coch^−/−^* control (*n* = 13).

## 5. Conclusions

Although cochlin is one of the most abundant proteins in the inner ear, its exact function remains unknown. Due to the presence of the LCCL domain in its protein structure, it is suggested that cochlin plays a role in our innate immune system by attracting neutrophils and monocytes. Noise exposure caused PTS in *Coch^+/+^* mice, while it only caused TTS in *Coch^−/−^* mice indicating that *Coch^−/−^* mice are less susceptible to NIHL. However, immunohistochemistry was unable to identify any differences in cell morphology and inner ear inflammation between both groups. Our results indicate that the absence of *Coch* has an influence on hearing thresholds after noise exposure, but this is not related to inner ear inflammation. Cochlin maintains the ion homeostasis and ECM structure of the inner ear due to its affinity for collagen II. Future research is needed to elucidate the role of cochlin as a potential mechanosensing molecule and to investigate whether changes in *Coch* expression may cause a disruption of this ion homeostasis and shear stress resulting in hearing loss.

## Figures and Tables

**Table 1:** Scoring (0–4) used to evaluate vestibular function through the VDR.

Score	Description
0	Normal behavior, normal reflex
1	Possibility of impaired behavior
2	Alteration in behavior, but limited
3	Important change in behavior
4	Extreme change in behavior

**Table 2:** Overview of antibodies used to stain the spiral ligament.

Primary Antibodies	Secondary Antibodies
Rat anti-COCH (Merck Milipore (MABF267), 1:200)	Goat anti-rat Fluor 555 (Jackson (112-076-062), 1:1000)
Rabbit anti-IBA1 (Wako (019-19741), 1:1000)	Goat anti-rabbit Fluor 555 (Abcam (ab6719), 1:1000)
Rat anti-F4/80 (AbD Serotec (MCA497GA), 1:250)	Goat anti-rat Fluor 555 (Jackson (112-076-062), 1:1000)
Rabbit anti-AQ1 (Sigma-Aldrich, 1:2000)	Donkey anti-rabbit Fluor 555 (Invitrogen (A31572) 1:1000)
Rabbit anti-CTGF (Abcam(ab6992), 1:500)	Donkey anti-rabbit Fluor 555 (Invitrogen (A31572) 1:1000)

## Data Availability

The data that support the findings of this study are available from the corresponding author upon reasonable request.

## Supplementary Materials

The following are available online at https://www.mdpi.com/article/10.3390/ijms222111549/s1, Table S1A: Mann–Whitney U test were performed to assess differences in DPOAE thresholds between Coch^+/+^ and Coch^−/−^ mice; Table S1B: Mann–Whitney U test were performed to assess differences in ABR thresholds between Coch^+/+^ and Coch^−/−^ mice; Table S2A: Linear mixed models (LMMs) were fitted for each DPOAE frequency separately to test for significant time × genotype interactions; Table S2B: Post-hoc tests to assess the difference in DPOAE thresholds between the different time points were performed if the main effect of time*WT and time*KO were significant (Table S1A); Table S3A: Linear mixed models (LMMs) were fitted for each ABR frequency separately to test for significant time × genotype interactions; Table S3B: Post-hoc tests to assess the difference in ABR thresholds between the different time points were performed if the main effect of time*WT and time*KO were significant (Table S3A); Table S4A: Linear mixed models (LMMs) were fitted for wave I amplitudes at 8000 Hz and 16,000 Hz separately to test for significant time × genotype interactions; Table S4B: Post-hoc tests to assess the difference in wave I amplitudes between the different time points were performed if the main effect of time*WT and time*KO were significant (Table S4A).
