# Supplementary material for: Cochlin Deficiency Protects Aged Mice from Noise-Induced Hearing Loss
Source: Int J Mol Sci. 2021 Oct 26;22(21):11549. doi: 10.3390/ijms222111549 (PMC8584124; doi:10.3390/ijms222111549)
Supplement: Supplementary file 1 [file ijms-22-11549-s001.zip › supplementary.pdf]

**Table S1A.** Mann–Whitney U test were performed to assess differences in DPOAE thresholds between *Coch*$^{+/+}$ and *Coch*$^{-/-}$ mice. *p*-values are reported. significant values ($p \leq 0.05$) are shown in bold.

| DPOAE frequency | 6 months | 12 months | 15 months | 24 months |
|---|---|---|---|---|
| 5 kHz | 0.97 | **0.01** | **<0.0001** | >0.99 |
| 6 kHz | 0.18 | **<0.0001** | **<0.0001** | 0.2867 |
| 7 kHz | 0.15 | **0.01** | **0.015** | 0.6417 |
| 8 kHz | 0.45 | **0.003** | 0.28 | 0.2554 |
| 9 kHz | 0.28 | **0.002** | 0.067 | 0.2085 |
| 10.5 kHz | 0.64 | **<0.0001** | 0.19 | 0.3607 |
| 12 kHz | 0.15 | 0.60 | 0.84 | 0.5392 |
| 14 kHz | 0.59 | **0.002** | 0.19 | 0.3007 |
| 16 kHz | 0.33 | **0.049** | 0.52 | 0.0782 |
| 18 kHz | >0.99 | **0.028** | >0.99 | **0.0165** |
| 21 kHz | 0.71 | **0.008** | 0.055 | 0.1696 |
| 24 kHz | 0.18 | **<0.0001** | **0.047** | 0.1932 |
| 28 kHz | 0.59 | **<0.0001** | **0.0022** | 0.1821 |
| 32 kHz | 0.64 | **0.017** | 0.057 | 0.7098 |

**Table S1B.** Mann–Whitney U test were performed to assess differences in ABR thresholds between *Coch*$^{+/+}$ and *Coch*$^{-/-}$ mice. *p*-values are reported. significant values ($p \leq 0.05$) are shown in bold.

| ABR frequency | 6 months | 12 months | 15 months | 24 months |
|---|---|---|---|---|
| 2000Hz | **0.0032** | **<0.0001** | 0.22 | >0.9999 |
| 4000Hz | 0.88 | **0.05** | 0.39 | 0.87 |
| 8000Hz | 0.74 | **0.0048** | 0.86 | 0.056 |
| 16000Hz | **0.0015** | **0.0006** | 0.81 | **0.016** |
| 32000Hz | 0.47 | **<0.0001** | **0.0008** | **0.0079** |

**Table S2A.** Linear mixed models (LMMs) were fitted for each DPOAE frequency separately to test for significant time x genotype interactions. Also main effects of time*KO and time*WT were calculated. Significant values ($p \leq 0.05$) are shown in bold.

| DPOAE frequency | Time*genotype interaction | Main effect Time*WT | Main effect Time*KO |
|---|---|---|---|
| 5 kHz | 0.057 | **0.0005** | **0.013** |
| 6 kHz | 0.175 | 0.064 | 0.24 |
| 7 kHz | 0.739 | **0.002** | 0.16 |
| 8 kHz | 0.326 | **0.0001** | **0.019** |
| 9 kHz | 0.537 | **$4.01*10^{-5}$** | **0.002** |
| 10.5 kHz | 0.690 | **0.0067** | **0.031** |
| 12 kHz | 0.403 | 0.38 | 0.088 |
| 14 kHz | 0.711 | **$3.01*10^{-6}$** | **0.0009** |
| 16 kHz | 0.765 | **$5.15*10^{-6}$** | **0.01** |
| 18 kHz | 0.339 | **$5.21*10^{-6}$** | **0.01** |
| 21 kHz | **0.028** | **$3.07*10^{-8}$** | **0.04** |
| 24 kHz | **$1.36*10^{5}$** | **$1.56*10^{-11}$** | **0.005** |
| 28 kHz | **0.0003** | **$2.36*10^{-10}$** | **0.007** |
| 32 kHz | **0.020** | **$2.32*10^{-8}$** | **$4.82*10^{-5}$** |

**Table S2B.** Post-hoc tests to assess the difference in DPOAE thresholds between the different time points were performed if the main effect of time*WT and time*KO were significant (Table S1A). Significant values (*p* ≤ 0.05) are shown in bold.

| DPOAE frequency | Coch-wildtype | | Coch- knockout | |
|---|---|---|---|---|
| | Baseline VS 48u post-noise | Baseline VS one-week post-noise | Baseline VS 48u post-noise | Baseline VS one-week post-noise |
| 5 kHz | **$3.71*10^{-5}$** | **0.006** | **0.029** | **0.008** |
| 6 kHz | NA | NA | NA | NA |
| 7 kHz | **0.0006** | **0.007** | NA | NA |
| 8 kHz | **$6.24*10^{-6}$** | **0.0003** | **0.012** | **0.049** |
| 9 kHz | **$2.58*10^{-6}$** | **$3.87*10^{-5}$** | **0.0012** | **0.0067** |
| 10.5 kHz | **0.0016** | 0.16 | **0.022** | 0.068 |
| 12 kHz | NA | NA | NA | NA |
| 14 kHz | **$1.44*10^{-7}$** | 0.405 | **$7.31*10^{-5}$** | 0.14 |
| 16 kHz | **$5.39*10^{-8}$** | **$9.04*10^{-5}$** | **0.0037** | 0.16 |
| 18 kHz | **$1.10*10^{-8}$** | **$1.64*10^{-5}$** | **0.0031** | 0.31 |
| 21 kHz | **$<1*10^{-10}$** | **$9.29*10^{-10}$** | **0.026** | 0.10 |
| 24 kHz | **$<1*10^{-10}$** | **$<1*10^{-10}$** | **0.0031** | **0.01** |
| 28 kHz | **$<1*10^{-10}$** | **$<1*10^{-10}$** | **0.0063** | **0.02** |
| 32 kHz | **$<1*10^{-10}$** | **$2.02*10^{-7}$** | **$1.32*10^{-5}$** | **$1.49*10^{-5}$** |

**Table S3A.** Linear mixed models (LMMs) were fitted for each ABR frequency separately to test for significant time x genotype interactions. Also main effects of time*KO and time*WT were calculated. Significant values (*p* ≤ 0.05) are shown in bold.

| ABR frequency | Time*genotype interaction | Main effect Time*WT | Main effect Time*KO |
|---|---|---|---|
| 2000Hz | **0.013** | **$3.62*10^{-10}$** | **0.0043** |
| 4000Hz | **0.003** | **$2.43*10^{-7}$** | 0.13 |
| 8000Hz | **$2.4*10^{-5}$** | **$1.41*10^{-9}$** | **0.019** |
| 16000Hz | **0.01** | **$1.31*10^{-14}$** | **$1.36*10^{-7}$** |
| 32000Hz | **$7.28*10^{-5}$** | **$2.39*10^{-10}$** | **$6.88*10^{-7}$** |

**Table S3B.** Post-hoc tests to assess the difference in ABR thresholds between the different time points were performed if the main effect of time*WT and time*KO were significant (Table S3A). Significant values (*p* ≤ 0.05) are shown in bold.

| ABR frequency | Coch-wildtype | | Coch- knockout | |
|---|---|---|---|---|
| | Baseline VS 48u post-noise | Baseline VS one week post-noise | Baseline VS 48u post-noise | Baseline VS one week post-noise |
| 2000Hz | **$<1*10^{-10}$** | **$1.17*10^{-8}$** | **0.0011** | 0.13 |
| 4000Hz | **$1.79*10^{-9}$** | **$3.94*10^{-7}$** | NA | NA |
| 8000Hz | **$<1*10^{-10}$** | **$<1*10^{-10}$** | **0.008** | 0.54 |
| 16000Hz | **$<1*10^{-10}$** | **$<1*10^{-10}$** | **$<1*10^{-10}$** | **$9.9*10^{-6}$** |
| 32000Hz | **$<1*10^{-10}$** | **$<1*10^{-10}$** | **$4.02*10^{-9}$** | 0.068 |

**Table S4A.** Linear mixed models (LMMs) were fitted for wave I amplitudes at 8000 Hzand 16000 Hz separately to test for significant time x genotype interactions. Also main effects of time*KO and time*WT were calculated. Significant values (*p* ≤ 0.05) are shown in bold.

| Wave I amplitude | Time*genotype interaction | Main effect Time*WT | Main effect Time*KO |
|---|---|---|---|
| 8000Hz | 0.574247 | **$5.03*10^{-5}$** | **0.007** |
| 16000Hz | 0.964111 | **0.001** | **0.0001** |

**Table S4B.** Post-hoc tests to assess the difference in wave I amplitudes between the different time points were performed if the main effect of time*WT and time*KO were significant (Table S4A). Significant values (*p* ≤ 0.05*)* are shown in bold.

| | **Coch-wildtype** | | **Coch- knockout** | |
|---|---|---|---|---|
| **Wave I amplitude** | **Baseline VS 48u post-noise** | **Baseline VS one week post-noise** | **Baseline VS 48u post-noise** | **Baseline VS one week post-noise** |
| 8000Hz | **0.0034** | **0.036** | **$6.66*10^{-6}$** | **$2.97*10^{-5}$** |
| 16000Hz | **$1.25*10\text{-}^{5}$** | **0.0017** | **0.00025** | **0.016** |
